# Analysis of the contribution of *FTO*, *NPC1, ENPP1, NEGR1, GNPDA2* and *MC4R* genes to obesity in Mexican children

**DOI:** 10.1186/1471-2350-14-21

**Published:** 2013-02-01

**Authors:** Aurora Mejía-Benítez, Miguel Klünder-Klünder, Loic Yengo, David Meyre, Celia Aradillas, Esperanza Cruz, Elva Pérez-Luque, Juan Manuel Malacara, Maria Eugenia Garay, Jesús Peralta-Romero, Samuel Flores-Huerta, Jaime García-Mena, Philippe Froguel, Miguel Cruz, Amélie Bonnefond

**Affiliations:** 1 Departamento de Genética y Biología Molecular, Centro de Investigación y de Estudios Avanzados del Instituto Politécnico Nacional, Mexico City, Mexico; 2 Community Health Research Department, Hospital Infantil de México Federico Gómez, Ministry of Health (SSA), Mexico City, Mexico; 3 CNRS-UMR8199, Lille Pasteur Institute, Lille, France; 4 Lille Nord de France University, Lille, France; 5 Department of Clinical Epidemiology and Biostatistics, Michael DeGroote Centre for Learning & Discovery, McMaster University, Hamilton, Canada; 6 Faculty of Medicine, University of San Luis Potosi, San Luis Potosi, Mexico; 7 Medical Science Department, University of Guanajuato, Guanajuato, Mexico; 8 Medical Research Unit in Biochemistry, UMAE Bernardo Sepúlveda, IMSS, Mexico City, Mexico; 9 Department of Genomics of Common Disease, School of Public Health, Imperial College London, Hammersmith Hospital, London, UK; 10 Present address: Inserm-U695, Paris 7 University, Paris, France

**Keywords:** Obesity, Mexican children, Single nucleotide polymorphism

## Abstract

**Background:**

Recent genome wide association studies (GWAS) and previous positional linkage studies have identified more than 50 single nucleotide polymorphisms (SNPs) associated with obesity, mostly in Europeans. We aimed to assess the contribution of some of these SNPs to obesity risk and to the variation of related metabolic traits, in Mexican children.

**Methods:**

The association of six European obesity-related SNPs in or near *FTO, NPC1, ENPP1, NEGR1, GNPDA2* and *MC4R* genes with risk of obesity was tested in 1,463 school-aged Mexican children (*N*_*cases*_ = 514; *N*_*controls*_ = 949). We also assessed effects of these SNPs on the variation of body mass index (BMI), fasting serum insulin levels, fasting plasma glucose levels, total cholesterol and triglyceride levels, in a subset of 1,171 nonobese Mexican children.

**Results:**

We found a significant effect of *GNPDA2* rs10938397 on risk of obesity (odds ratio [OR] = 1.30; *P* = 1.34 × 10^-3^). Furthermore, we found nominal associations between obesity risk or BMI variation and the following SNPs: *ENPP1* rs7754561, *MC4R* rs17782313 and *NEGR1* rs2815752. Importantly, the at-risk alleles of both *MC4R* rs17782313 and *NPC1* rs1805081 showed significant effect on increased fasting glucose levels (β = 0.36 mmol/L; *P* = 1.47 × 10^-3^) and decreased fasting serum insulin levels (β = −0.10 μU/mL; *P* = 1.21 × 10^-3^), respectively.

**Conclusion:**

Our present results suggest that some obesity-associated SNPs previously reported in Europeans also associate with risk of obesity, or metabolic quantitative traits, in Mexican children. Importantly, we found new associations between *MC4R* and fasting glucose levels, and between *NPC1* and fasting insulin levels.

## Background

Obesity and associated comorbidities (such as cardiovascular diseases, type 2 diabetes, musculoskeletal disorders and some cancers) represent a major public health problem that, in recent years, has reached epidemic proportions. In Mexico, obesity is the most common nutritional disorder of children, and the prevalence of this disease has increased alarmingly over the past decade: recent data from ENSA 2006 (‘*Encuesta Nacional de Salud*’, *i.e.* The National Health Census) reported an increase in overweight and obesity from 18% in 1999 to 26% in 2006, in young Mexican children (between 5 and 11 years old) [1]. Overweight in childhood or adolescence is a risk factor for overall mortality in adulthood [2].

The prevalence of obesity varies across populations and it is noteworthy that the Mexican population has been disproportionally affected. Although environmental changes (linked to the ‘westernization’ of ways of life) can explain the increase in prevalence of obesity on a global level, individual variation of body mass index (BMI) persists in the same environment, and the heritability of this trait is very high. Indeed, heritability estimates of BMI range between 40% and 70% according to studies [3]. The high genetic susceptibility to obesity (and both insulin resistance and type 2 diabetes) in the Mexican population may be ascribed to the American Indian heritage [4].

To date, genome wide association studies (GWAS) and linkage studies, mostly performed in European adult populations, have identified more than 50 loci associated with obesity or BMI [5-10]. However, replication attempts have yielded inconsistent outcomes [11-13]. In particular, replication in other populations is not obvious, and several factors (such as ethnic differences in linkage disequilibrium patterns, ethnic-specific associations, gene × environment interactions) may puzzle the picture. Furthermore, very few studies have been performed in children presenting with severe obesity in non-European populations.

In the present study, we aimed to perform a follow-up replication study including sixEuropean obesity-associated genetic variants, in Mexican children.

## Methods

### Study participants

In the present study, we analyzed 1,685 children (aged 6 to 12 years) of Mexican origin, from five different states of Mexico (San Luis Potosí, Queretaro, Tijuana, Guanajuato and Mexico city), who were randomly selected and invited to participate in a cross-sectional study between 2007 and 2011 from public and private schools. Child assent was obtained and parents provided written informed consent. We collected data from the children and parent or legal guardian per child by direct questioning.

Participants were scheduled for clinical laboratory evaluation following a 12 h overnight fasting. Blood samples were drawn to assess levels of fasting glucose, fasting insulin, total cholesterol and triglycerides levels. Biochemical variables were measured using an ILab 350 Clinical Chemistry System (Instrumentation Laboratory IL). Weight was measured with a digital scale (Seca) and height was measured with a portable stadiometer (Seca 225). BMI was calculated and classified according to the ‘Centers for Disease Control and Prevention 2000’ (CDC 2000) reference [14]. CDC 2000 growth charts are based on 5 U.S nationally representative surveys conducted between 1963 and 1994, in which Mexican American children were included [14]. According to those growth charts, for ages 2 to 20 years, overweight was defined as a BMI-for-age between the 85^th^ and 95^th^ percentiles, while obesity was defined when BMI-for-age was higher than the 95^th^ percentile [14,15]. Insulin resistance was defined as: homeostasis model assessment of insulin resistance (HOMA-IR = [(Fasting glucose _(mg/dL)_) (Fasting insulin _(μU/mL)_)]/405) ≥ 3.4 (that is the 90^th^ percentile of HOMA-IR in a population of healthy Mexican children [16,17]).

The study was approved by the local ethics committees (‘Instituto Mexicano del Seguro Social’, *i.e.* the Mexican national health service; reference number: 2011-000-079) and is in compliance with the Helsinki Declaration.

### SNPs Selection and genotyping

To achieve a power of at least 50%, with an odds ratio of 1.25, we only selected SNPs with minor allele frequencies ≥ 15% in the Mexican population according to the HapMap database. These SNPs were identified by GWAS or meta-analyses of GWAS in European populations [6,7,9,10]. Furthermore, we selected a SNP that was significantly associated with childhood obesity in a French population, according to a linkage association study [8]. The expected statistical power per SNP (estimated by Quanto software) is reported in Additional file 1: Table S1.

Genomic DNA was isolated from peripheral blood white cells using a QIAamp DNA kit (Qiagen), purity and integrity was verified by 260/280 nm measurements and by electrophoresis in 0.8% agarose gels, stained with ethidium bromide.

All DNA samples were genotyped for the *MC4R* rs17782313, *NEGR1* rs2815752, *ENPP1* rs7754561, *NPC1* rs1805081, *GNPDA2* rs10938397 and *FTO* rs1421085 using TaqMan assays on an ABI7900 system, following the manufacturer’s protocol (Applied Biosystems). The genotype success rate was at least 98%, and no deviation (*P* ≥ 0.01) from Hardy-Weinberg equilibrium was observed in our population. Thirty duplicate quality control samples were included and were genotyped with 100% concordance.

### Statistical analysis

The effect of SNPs on obesity status was assessed using a logistic regression model adjusted for age, gender and Mexican state (1: San Luis Potosí, 2: Queretaro, 3: Tijuana, 4: Guanajuato and 5: Mexico city), under an additive model. We also analyzed the effect of SNPs on several metabolic quantitative traits (BMI, fasting serum insulin, fasting plasma glucose, total cholesterol and triglycerides) using linear regressions under an additive model adjusted for age, gender and Mexican state. Data for fasting serum insulin and triglycerides were logarithmically transformed before statistical analysis. By applying Bonferroni correction, a significant p-value has been considered when below 1.4 × 10^-3^ (0.05/36) and a p-value between 0.05 and 1.4 × 10^-3^ has been considered as nominally significant. All statistical analyses were performed using the SPSS software (version 14.0).

## Results

From an initial sample of 1,685 children (between 6 and 12 years old), we extracted 949 lean and 514 obese children (see clinical characteristics in Table 1) for the case–control study, and 1,171 nonobese children (with BMI < 95^th^ percentile) for the study of metabolic quantitative traits including BMI, fasting serum insulin, fasting plasma glucose, total cholesterol and triglycerides. As expected, obese children exhibited higher values of insulin and cholesterol levels (*P* ≤ 0.001) compared to lean children. Insulin resistance was found in 45% of obese children.

**Table 1:** Clinical characteristics of lean and obese Mexican children

	**Lean children**	**Obese children**	
**Characteristics**	**BMI <85pc**	**BMI ≥95pc**	P*
	***N*** **= 949**	***N*** **= 514**	
Female (%)	48.4	43.6	0.04
Age (Years)	9.5 ± 1.9	9.5 ± 1.8	0.383
**Anthropometric**			
Weight (kg)	32.6 ± 7.8	49.7 ± 13.4	<0.001
Height (cm)	134.3 ± 11.8	139.6 ± 11.7	<0.001
BMI (kg/m^2^)	17.7 ± 1.4	25.0 ± 3.4	<0.001
BMI (percentile)	65.8 ± 11.2	97.6 ± 1.4	<0.001
**Metabolic factors**			
Fasting Insulin (μU/mL)	8.6 ± 5.9	14.1 ± 9.4	<0.001
Fasting glucose (mmol/L)	4.7 ± 0.6	4.9 ± 0.6	0.0011
Total Cholesterol (mg/dL)	151.8 ± 29.6	160.1 ± 35.1	<0.001
Triglycerides (mg/dL)	88.5 ± 38.2	126.6 ± 67.6	<0.001

Data are means ± standard deviation.

*P-value according to Student’s *t* test comparing lean and obese children.

*BMI*, body mass index; *pc*, percentile.

In both studies, we genotyped six SNPs that are known to be associated with risk of obesity in European populations: rs17782313 near *MC4R*[6]; rs2815752 near *NEGR1*[7]; rs7754561 near *ENPP1*[8]; rs1805081 in exon 6 of *NPC1*[9]; rs10938397 near *GNPDA2*[7] and rs1421085 in intron 1 of *FTO*[10].

We identified a significant contribution of *GNPAD2* rs10938397 to risk of obesity (G risk allele [in European populations]; odds ratio_[95% confidence interval]_ [OR] = 1.30_[1.11;1.53]_; *P* = 1.34 × 10^-3^; Table 2). Furthermore, we found nominal association between risk of obesity and both *ENPP1* rs7754561 (G risk allele; OR = 0.84_[0.72;0.97]_; *P* = 0.020; Table 2) and *MC4R* rs17782313 (C risk allele; OR = 1.40_[1.06;1.83]_; *P* = 0.016; Table 2). This last *MC4R* SNP also showed a nominal effect on BMI (C risk allele; β_[standard error]_ = 0.41_[0.16]_ kg/m^2^; *P* = 0.012; Table 3). Moreover, we identified a nominal association between *NEGR1* rs2815752 and BMI (A risk allele; β = 0.24_[0.10]_ kg/m^2^; *P* = 0.019; Table 3). Of note, we did not find any significant association between risk of obesity (or BMI variation) and either *NPC1* rs2815752 or *FTO* rs1421085 (Tables 2 and Table 3).

**Table 2:** **Association between SNPs *MC4R *rs17782313, *NEGR1 *rs2815752, *ENPP1 *rs7754561, *NPC1 *rs1805081, *GNPDA2 *rs10938397 and *FTO *rs1421085, and obesity in Mexican children**

**Locus**	**SNP**	RA*	**Cases/**	***N***	**RAF**	**OR**	***P***	
			**Controls**		**(%)**	**[95% CI]**		
			Lean children	949	74.4	-	-	
***NEGR1***	rs2815752	A						
			Obese children	514	77.6	1.18 [0.99;1.41]	0.068	
			Lean children	949	74.6	-	-	
***NPC1***	rs1805081	A						
			Obese children	514	74.2	1.02 [0.85;1.21]	0.845	
			Lean children	949	**48.6**	-	-	
***ENPP1***	rs7754561	G						
			Obese children	514	**44.2**	**0.84 [0.72;0.97]**	**0.020**	
			Lean children	949	**7.3**	-	-	
***MCR4***	rs17782313	C						
			Obese children	514	**9.8**	**1.40 [1.06;1.83]**	**0.016**	
			Lean children	949	**32.2**	-	-	
***GNPDA2***	rs10938397	G						
			Obese children	514	**38.0**	**1.30 [1.11;1.53]**	**1.34 × 10**^**-3**^	
			Lean children	949	19.1	-	-	
***FTO***	rs1421085	C						
			Obese children	514	21.1	1.13 [0.93;1.38]	0.228	

*We noted the risk-alleles reported in European populations.

*RA*, risk-allele; *RAF*, risk-allele frequency; *OR*, odds ratio; *CI*, confidence interval; *P*, P-Value.

**Table 3:** **Association between SNPs *MC4R *rs17782313, *NEGR1 *rs2815752, *ENPP1 *rs7754561, *NPC1 *rs1805081, *GNPDA2 *rs10938397 and *FTO *rs1421085, and metabolic quantitative traits in 1,171 nonobese Mexican children**

		**BMI**	**Fasting serum insulin**		**Fasting plasma glucose**		**Total cholesterol**		**Triglycerides**	
Locus SNP RA*	***β *(SE)**	***P***	***β *(SE)**	***P***	***β *(SE)**	***P***	***β *(SE)**	***P***	***β *(SE)**	***P***
***NEGR1 r*s2815752 A**	**0.24**	**0.019**	0.01	0.611	0.07	0.353	2.14	0.139	0.01	0.425
	**(0.10)**		(0.03)		(0.07)		(1.44)		(0.02)	
***NPC1 *rs1805081 A**	−0.16	0.126	**−0.10**	**1.21 × 10**^**-3**^	−0.08	0.278	2.22	0.144	−0.003	0.884
	(0.11)		**(0.03)**		(0.07)		(1.52)		(0.02)	
***ENPP1 *rs7754561 G**	−0.11	0.209	0.004	0.873	0.01	0.905	2.20	0.087	0.004	0.827
	(0.09)		(0.03)		(0.06)		(1.29)		(0.02)	
***MCR4 *rs17782313 C**	**0.41**	**0.012**	0.04	0.362	**0.36**	**1.47 × 10**^**-3**^	2.71	0.247	0.02	0.410
	**(0.16)**		(0.05)		**(0.11)**		(2.34)		(0.03)	
***GNPDA2 *rs10938397 G**	0.10	0.288	0.04	0.126	0.10	0.132	1.90	0.176	0.03	0.076
	(0.10)		(0.03)		(0.07)		(1.40)		(0.02)	
***FTO *rs1421085 C**	−0.17	0.171	−0.06	0.064	−0.14	0.111	**3.70**	**0.032**	0.006	0.778
	(0.12)		(0.04)		(0.09)		**(1.72)**		(0.02)	

Data for fasting serum insulin and triglycerides were logarithmically transformed before statistical analysis. Units are: BMI in kg/m^2^, fasting serum insulin in μU/mL, fasting plasma glucose in mmol/L, total cholesterol in mg/dL and triglycerides in mg/dL.

*We noted the risk-alleles reported in European populations.

*RA*, risk-allele; *SE*, standard error; *β*, effect-size; *P*, P-value; *BMI*, body mass index.

In our study of quantitative traits, we found a significant association between *NPC1* rs1805081 and fasting serum insulin levels (A risk allele; β = −0.10_[0.03]_ μU/mL; *P* = 1.21 × 10^-3^; Table 3). We also identified an effect of the obesity risk allele of *MC4R* rs17782313 on increased fasting plasma glucose levels (C risk allele; β = 0.36_[0.11]_ mmol/L; *P* = 1.47 × 10^-3^; Table 3). A trend of association was found between *FTO* rs1421085 and both total cholesterol levels (C risk allele; β = 3.70_[1.72]_ mg/dL; *P* = 0.032; Table 3) and fasting serum insulin levels (C risk allele; β = −0.06_[0.04]_ μU/mL; *P* = 0.064; Table 3).

## Discussion

From our study based on obese and nonobese Mexican children, we found a significant contribution of the minor allele of *GNPDA2* rs10938397 to increased risk of obesity. The association between *GNPDA2* and BMI had firstly been identified in a meta-analysis of several European GWASs performed by the GIANT consortium [7]. Subsequently, the association signal has been confirmed in other populations: in adults from East-Asia [18,19] and in Chinese children [20]. To our knowledge, the present study is the first confirmation of the *GNPDA2* association signal with risk of obesity in the Mexican population.

Furthermore, we found nominal associations between risk of obesity or BMI and the following SNPs: *ENPP1* rs7754561, *MC4R* rs17782313 and *NEGR1* rs2815752. Of note, the risk alleles for obesity or increased BMI were the same between Europeans and Mexicans, except for the risk allele of *ENPP1* rs7754561 (in Europeans) that showed a protective effect in Mexicans. Although the association between both *MC4R* and *NEGR1* and risk of obesity has been confirmed in a plethora of studies and populations, the *ENPP1* association signal with obesity is more controversial [21]. To our knowledge, no other studies demonstrate a protective role of the risk allele of *ENPP1* rs7754561. Recently, it has been shown that *ENPP1* overexpression in human adipocyte cell lines resulted in defective adipocyte maturation [22]. If confirmed in other Mexican populations, the protective effect of the *ENPP1* variant may be due to a loss-of-function of the protein.

We did not find any significant contribution of either *NPC1* rs2815752 or *FTO* rs1421085 to obesity or BMI variation. Therefore, the *FTO* association signal with obesity which was found in Mexican adults by Villalobos-Comparán and colleagues [23], was not confirmed in children. Thus, the obesogenic effect of *FTO* would occur later in the Mexican population than in the Europeans [9], but as we lack some statistical power, additional genetic studies on Mexican children would be needed.

Importantly, we identified a significant effect of the risk allele of *NPC1* rs1805081 on decreased fasting serum insulin levels. *NPC1* encodes Niemann Pick disease type C1 protein that mediates intracellular cholesterol trafficking via binding of cholesterol to its N-terminal domain. Very recently, Jelinek and colleagues have reported that *Npc1* haploinsufficiency developed abnormal metabolic features (including hyperinsulinemia) and increased susceptibility to weight gain in mice [24]. We could suspect that the variant may have a gain-of-function effect in Mexican children. Of note, we did not report any association between the *NPC1* variant and obesity or BMI variation, and this association signal remains quite controversial according to population studies [20,25,26].

Furthermore, we identified an effect of the risk allele of *MC4R* variant and increased fasting plasma glucose levels. To our knowledge, no previous study has shown this association. However, two recent large meta-analyses of GWAS identified a significant association between the *MC4R* locus and type 2 diabetes risk, in European and Asian populations [27,28]. Of note, our present association with fasting plasma glucose levels remained significant after adjustment for BMI (data not shown). Altogether, these findings would suggest a potential effect of *MC4R* polymorphisms on decreased pancreatic beta-cell function.

## Conclusion

In summary, in our sample of Mexican children, we replicated four European obesity-related genes (*GNPDA2*, and nominally, *NEGR1*, *MC4R* and *ENPP1*) in the same direction of effect as previous findings, except for *ENPP1*. Interestingly, we found two novel association signals: between *NPC1* variant and fasting serum insulin levels, and between *MC4R* variant and fasting plasma glucose levels. These findings should deserve some confirmatory studies in other Mexican populations.

It is noteworthy that these well established genetic associations with obesity explain very little of the genetic risk for pediatric phenotype, suggesting the existence of additional loci whose number and effect size remain unknown, which guarantee intense additional investigations in the near future.

## Abbreviations

β: Effect-size; BMI: Body mass index; CI: Confidence interval; GWAS: Genome-wide association study; HOMA-IR: Homeostasis model assessment of insulin resistance; OR: Odds ratio; P: P-value; pc: Percentile; RA: Risk-allele; RAF: Risk-allele frequency; SE: Standard error; SNP: Single nucleotide polymorphism.

## Competing interests

The authors declare no competing financial interests.

## Authors’ contributions

AMB performed the genotyping, and contributed to statistical analyses and the writing of the paper; AB wrote the paper; DM, JGM, PF and MC revised the paper and contributed to discussion; LY and AB performed the statistical analyses; AMB, MKK, CA, EC, EPL, JMM, MEG, JPR, SFH, JGM and MC contributed to recruitment and clinical data of the Mexican children. All the authors approved the final version of the submitted draft.

## Pre-publication history

The pre-publication history for this paper can be accessed here:

http://www.biomedcentral.com/1471-2350/14/21/prepub

## Supplementary Material

Additional file 1: Table S1 Expected statistical power for reaching an odds ratio of 1.25 in the present obesity case-control study (*N*_*cases*_ = 514; *N*_*controls*_ = 949). Click here for file
