# Supplementary material for: Analysis of the contribution of FTO, NPC1, ENPP1, NEGR1, GNPDA2 and MC4R genes to obesity in Mexican children
Source: BMC Med Genet. 2013 Feb 1;14:21. doi: 10.1186/1471-2350-14-21 (PMC3577489; doi:10.1186/1471-2350-14-21)
Supplement: Additional file 1: Table S1 — Expected statistical power for reaching an odds ratio of 1.25 in the present obesity case-control study (Ncases = 514; Ncontrols = 949). [file 1471-2350-14-21-S1.docx]

**Supplementary Table 1.** Expected statistical power for reaching an odds ratio of 1.25 in the present obesity case-control study (*N_cases_*=514; *N_controls_*=949)

| **SNP** | **RAF*** | **Statistical Power** |
| --- | --- | --- |
| MC4R rs17782313 | 0.15 | 53% |
| NEGR1 rs2815752 | 0.73 | 67% |
| ENPP1 rs7754561 | 0.46 | 78% |
| NPC1 rs1805081 | 0.82 | 55% |
| GNPDA2 rs10938397 | 0.24 | 66% |
| FTO rs1421085 | 0.18 | 55% |

*Risk allele frequency in the Mexican population, according to HapMap.

The odds ratio of 1.25 was chosen according to published obesity case-control studies with the same size [1, 2].

**Supplementary references**

1. Rouskas K, Kouvatsi A, Paletas K, Papazoglou D, Tsapas A, Lobbens S, Vatin V, Durand E, Labrune Y, Delplanque J *et al*: **Common variants in FTO, MC4R, TMEM18, PRL, AIF1, and PCSK1 show evidence of association with adult obesity in the Greek population**. *Obesity (Silver Spring)* 2012, **20**(2):389-395.

2. Ghoussaini M, Stutzmann F, Couturier C, Vatin V, Durand E, Lecoeur C, Degraeve F, Heude B, Tauber M, Hercberg S *et al*: **Analysis of the SIM1 contribution to polygenic obesity in the French population**. *Obesity (Silver Spring)* 2010, **18**(8):1670-1675.
